# The root‐invading pathogen *Fusarium oxysporum* targets pattern‐triggered immunity using both cytoplasmic and apoplastic effectors

**DOI:** 10.1111/nph.16618

**Published:** 2020-05-18

**Authors:** Nico Tintor, Misha Paauw, Martijn Rep, Frank L. W. Takken

**Affiliations:** ^1^ Molecular Plant Pathology SILS University of Amsterdam PO Box 94215 1090 GE Amsterdam the Netherlands

**Keywords:** *Arabidopsis thaliana*, PAMP‐triggered immunity, root, RXLR motif, vascular disease, wilt disease

## Abstract

- Plant pathogens use effector proteins to promote host colonisation. The mode of action of effectors from root‐invading pathogens, such as *Fusarium oxysporum* (*Fo*), is poorly understood. Here, we investigated whether *Fo* effectors suppress pattern‐triggered immunity (PTI), and whether they enter host cells during infection.
- Eight candidate effectors of an Arabidopsis‐infecting *Fo* strain were expressed with and without signal peptide for secretion in *Nicotiana benthamiana* and their effect on flg22‐triggered and chitin‐triggered reactive oxidative species (ROS) burst was monitored. To detect uptake, effector biotinylation by an intracellular Arabidopsis‐produced biotin ligase was examined following root infection.
- Four effectors suppressed PTI signalling; two acted intracellularly and two apoplastically. Heterologous expression of a PTI‐suppressing effector in Arabidopsis enhanced bacterial susceptibility. Consistent with an intracellular activity, host cell uptake of five effectors, but not of the apoplastically acting ones, was detected in *Fo*‐infected Arabidopsis roots.
- Multiple *Fo* effectors targeted PTI signalling, uncovering a surprising overlap in infection strategies between foliar and root pathogens. Extracellular targeting of flg22 signalling by a microbial effector provides a new mechanism on how plant pathogens manipulate their host. Effector translocation appears independent of protein size, charge, presence of conserved motifs or the promoter driving its expression.

## Introduction

Plant pathogens cause severe damage in agriculture, resulting in average yield losses of 20% worldwide (Strange & Scott, 2005; Fisher *et al.*, 2012). Central to their parasitic lifestyle is the ability to manipulate their host. Bacteria, fungi and oomycetes, but also nematodes and insects, employ small, secreted proteins called effectors for this manipulation (Lo Presti *et al.*, 2015; Toruno *et al.*, 2016; Uhse & Djamei, 2018). Revealing the host processes targeted by effectors is key to understanding the virulence strategy of a pathogen.

Plants employ a two‐layered immune system to ward off biotic threats. Recognition of microbe‐derived molecules (called PAMPs, for pathogen‐associated molecular patterns) triggers activation of a generic defence response (Cook *et al.*, 2015; Couto & Zipfel, 2016; Gust *et al.*, 2017). Classical PAMPs are bacterial flagellin (and its derived peptide flg22) and fungal chitin (Boller & Felix, 2009; Shinya *et al.*, 2015). PAMP perception is mediated by cell‐surface localised receptors, such as the flagellin receptor FLS2. Upon flg22 binding, FLS2 associates with the co‐receptor BAK1 and forms a signalling complex (Schulze *et al.*, 2010). Chitin perception in Arabidopsis depends on the receptor‐like protein and ‐kinase (RLP/RLK) LYK5 and CERK1 (Shinya *et al.*, 2015). PAMP recognition induces a series of physiological events, including changes in ion fluxes, production of ROS, activation of protein kinases and transcriptional reprogramming. Altogether, this pattern‐triggered immunity (PTI) limits colonisation by most potential pathogens. The second layer of plant immunity can be activated upon recognition of pathogen‐produced effectors by resistance genes that typically encode either intracellular NB‐LRR or cell‐surface RLK/RLP‐type receptors. Effector perception by resistance proteins generally induces a strong immune response, often involving host cell death (Jones & Dangl, 2006).

Bacterial effectors frequently target PTI by interfering with intracellular signal transduction (Dou & Zhou, 2012). Many oomycete effectors also act inside plant cells to repress PTI, although the underlying mechanisms are less well understood (Fabro *et al.*, 2011; Zheng *et al.*, 2014). By contrast, known fungal effectors typically interfere with PAMP perception rather than PTI signalling. They do so by, for example, binding chitin or beta‐glucan fragments in the apoplast, hiding them for the immune receptors (Lo Presti *et al.*, 2015; Wawra *et al.*, 2016). Known intracellular functions of fungal effectors involve manipulation of metabolic processes, or of transcriptional regulators (Djamei *et al.*, 2011; Plett *et al.*, 2014; Tanaka *et al.*, 2014; Wessling *et al.*, 2014). Recent evidence has indicated that fungal effectors can repress intracellular PTI signalling as well (Di *et al.*, 2017; Irieda *et al.*, 2019; Navarrete *et al.*, 2019), although it remains to be shown whether this represents a generic strategy for fungi.

To target intracellular PTI signalling, effectors have to enter plant cells. Whereas the delivery of bacterial effectors via the type III secretion system is relatively well understood, how eukaryotic effectors reach the plant cytoplasm remains enigmatic (Petre & Kamoun, 2014). Translocated oomycete effectors carry well defined motifs close to their N‐terminus, notably the RxLR–dEER and the LFLAK motif, that are critical for targeting them into host cells. Although the underlying mechanism is controversial (Whisson *et al.*, 2007; Kale *et al.*, 2010; Schornack *et al.*, 2010; Wawra *et al.*, 2017), these motifs are useful for bioinformatics‐assisted identification of potential cytoplasmic effectors in these pathogens. In contrast to oomycete effectors, conserved sequence motifs have not yet been described for cytoplasmic fungal effectors, except for an Y/F/WxC motif specific for a family of powdery mildew effectors (Godfrey *et al.*, 2010). Thus, discriminating cytoplasmic from apoplastic fungal effectors is currently not possible based on sequence analysis, and the properties explaining effector translocation into host cells remain to be elucidated.

The concept of PTI and its suppression by intracellularly acting effectors is largely based on the study of foliar pathogens. Roots are exposed to both beneficial and pathogenic microbes, a condition that has likely shaped the immune system in roots (De Coninck *et al.*, 2015; Hacquard *et al.*, 2017; Zhou *et al.*, 2020). Currently, it is poorly understood which type of defence responses are activated in roots, and whether root pathogens use similar mechanisms for their suppression as those employed in aboveground interactions. *Fo* is a widespread root coloniser that can cause disease on more than 120 different plant species, many of agricultural importance (Edel‐Hermann & Lecomte, 2019). Whereas colonisation of the root surface and the cortex is generally symptomless, pathogenic isolates invade the vasculature and grow upwards in the xylem vessels causing disease (Michielse & Rep, 2009). Individual strains have a narrow host range that is potentially determined by their effector repertoires (van Dam *et al.*, 2016). Initially, a set of 14 candidate effectors from a tomato‐infecting strain (*Fo* f.sp. *lycopersici*; *Fol*) was identified by proteomic analysis of the xylem sap of infected tomato plants, and hence these are named Secreted in xylem (Six) proteins. They all carry a signal peptide, are relatively small (<300 amino acids) and the encoding genes locate in the genome close to a transposable element (Schmidt *et al.*, 2013; Gawehns *et al.*, 2015). Three of them are recognised as avirulence factors: Six3 (Avr2) is recognised by an NB‐LRR, whereas Six1 (Avr3) and Six4 (Avr1) are recognised by an RLK and an RLP, respectively (Houterman *et al.*, 2009; Catanzariti *et al.*, 2015; Catanzariti *et al.*, 2017). Even though several *Fo* effectors were shown to contribute to virulence, an underlying mechanism has been proposed for only two of them: Avr2 from a tomato‐infecting strain (*Fo* f.sp. *lycopersici*; *Fol*) targets PTI, whereas Six5 promotes cell‐to‐cell movement of Avr2 (Di *et al.*, 2017; Cao *et al.*, 2018). Thus, it remains an intriguing question how this hemi‐biotrophic pathogen uses its effector repertoire for successful root and xylem colonisation.

Here we report the systematic identification, and characterisation, of candidate effectors from an Arabidopsis‐infecting *Fo* strain, Fo5176. To reveal their cellular site of action, effectors were targeted to either the cytosol or the apoplast of *N. benthamiana* leaves and their effect on a typical PTI response was assessed. In addition, translocation of effectors into host cells during Arabidopsis root colonisation was monitored. Our work identifies PTI as a common target for *Fo* effectors. Furthermore, effector translocation into host cells is not determined by their promoter or untranslated region (UTR) sequences, nor by general protein properties such as size and charge.

## Materials and Methods

### Plant material and fungal strains

Arabidopsis Col‐0 plants expressing the bacterial biotin ligase BirA have been described previously (Deal & Henikoff, 2011), as have been the *Fusarium oxysporum* isolate Fo5176 (Thatcher *et al.*, 2009) and the Fo5176 pSix1::GFP reporter strain (Kesten *et al.*, 2019).

### Plasmid construction

For producing Avi‐tagged effectors in Fo5176, the binary vector pRW2h (Houterman *et al.*, 2008) was modified as follows: an oligonucleotide (oligonucleotide sequences are listed in Supporting Information Table S1) containing the coding sequences of a single HA unit followed by the AviTag™ was inserted into a *Xba*I‐ and *Bgl*III‐digested vector. The oligonucleotide contained a single *Asc*I site preceding the HA–AviTag™. Next, the *FEM1* promoter was replaced by *c.* 1 kb promoter fragment directly 5′ of the Fo5176 *SIX1* gene, using *Hind*III and *Xba*I sites. The obtained vector was named pRW2h‐Six1p‐HB.

For expressing the effector genes *in planta*, the binary vector pBIN61 (SLDB3104; Tameling *et al.*, 2010) was modified as follows: restriction sites were added to the aforementioned HA–AviTag oligonucleotide, which was then inserted into the *Xba*I and *Xma*I sites of SLDB3104. The obtained vector was named pBIN61‐HB.

The coding sequence of effector genes was amplified by PCR, using as template cDNA obtained from *Fo*‐inoculated Arabidopsis seedling roots (to be described later). The primers introduced *Asc*I restriction sites to both ends of the PCR product, allowing its ligation into the *Asc*I site of pRW2h‐Six1p‐HB and pBIN61‐HB, respectively. The obtained plasmids allowed production of effectors that are C‐terminally fused to the HA–AviTag.

### *Agrobacterium*‐mediated transient transformation of *N. benthamiana*

*Agrobacterium tumefaciens* strain GV3101 was transformed with binary vectors and used for transient transformation of *N. benthamiana* as described previously (Ma *et al.*, 2012) with following modifications: Overnight cultures were resuspended in infiltration medium (10 mM MgCl_2_, 10 mM MES, 200 µM acetosyringone, pH 5.8) and infiltrated into leaves of *c.* 4‐wk‐old *N. benthamiana* plants at an OD_600_ of 0.5. A strain containing the silencing suppressor P19 was co‐infiltrated at an OD_600_ of 0.5.

### Generation of stable transgenic Arabidopsis

Arabidopsis Col‐0 plants expressing *BirA* were transformed with agrobacteria containing the binary vector pBIN‐dspFoa2‐HB using the floral dip method. Transformants were selected on medium containing 40 mg l^−1^ kanamycin. Single insertion lines were selected according to segregation ratio analysis, and putative transformants were confirmed by PCR and by Western blot. Two independent transformants differing in dspFoa2 accumulation (named 5‐1 and 6‐6) were selected. As the Col‐0 BirA line was transformed with dspFoa2, this line was also used as the wild‐type control in all experiments.

### Reactive oxidative species (ROS) assay

ROS production was measured with a luminol/peroxidase‐based assay as described previously (Felix *et al.*, 1999). Briefly, *N. benthamiana* leaf discs (5 mm diameter) were collected 2 d after *Agrobacterium* infiltration and floated overnight on deionised water (MQ) in a Petri dish. The next morning, leaf discs were transferred to 96‐well plates (Perkin Elmer, Waltham, MA, USA, white) containing 100 µl water per well, and immediately before measurement, 100 µl of a luminol/peroxidase solution was added to each well. Final concentrations were 250 µM luminol (Sigma), 10 µg ml^−1^ peroxidase (Sigma) and 200 nM flg22. To measure chitin‐induced ROS production, a final concentration of 10 µM chitin hexaose (Elicityl SA, Crolles, France) and 250 µM luminol L‐012 were used. Luminescence was recorded using 2‐min intervals for 1 h using a plate reader (Synergy H1; BioTek, Winooski, VT, USA). To measure ROS in Arabidopsis, leaf discs from 5–6‐wk‐old plants were collected, and the experiment was performed similarly as for *N. benthamiana*, except that luminol L‐012 was used for both flg22 and chitin assays.

### Mitogen‐activated protein kinase assay

Leaf discs from 5–6‐wk‐old Arabidopsis plants were floated on MQ water overnight and then exposed to 100 nM flg22 or 10 µM chitin hexaose for 0, 5 or 15 min. Proteins were isolated from 10 mg plant material as described (Flury *et al.*, 2013) and separated by sodium dodecyl sulfate polyacrylamide gel electrophoresis (SDS‐PAGE) using 10% gels, followed by Western blotting. The primary antibody (anti‐p44/p42 MAPK, monoclonal D13.14.4E; Cell Signaling Technology, Leiden, Netherlands) was used at a 1 : 6000 dilution, and the secondary antibody (goat‐anti‐rabbit; Pierce, Appleton, WI, USA, 31460) at a 1 : 10 000 dilution. Membranes were developed using the ECL plus kit from Thermo Scientific (Waltham, MA, USA).

### *Pseudomonas syringae* infection assay

Arabidopsis inoculation with *P. syringae* pathovar tomato (*Pst*) DC3000 was performed as described previously (Saijo *et al.*, 2009), with following modifications: 5–6‐wk‐old plants were spray inoculated with bacteria at an OD_600_ of 0.2, and bacterial titres were determined at 4 d post inoculation (dpi).

### Transformation of *F. oxysporum*

Stable transformants of Fo5176 were generated by *Agrobacterium*‐mediated transformation as described previously (Takken *et al.*, 2004). Successful transformants were selected based on hygromycin resistance. For each construct, at least five independent transformants were used for inoculation of Arabidopsis seedlings, followed by Western blotting to detect accumulation of tagged effectors in the infected seedlings.

### Arabidopsis inoculation with *Fo*

Fo51756 was grown on Czapek‐Dox‐Agar medium. To obtain spores, four agar plugs (each *c.* 0.5 × 0.5 cm) containing fungal mycelium were added to 100 ml liquid medium (3% sucrose, 100 mM KNO_3_ and 0.17% yeast nitrogen base) and incubated on a shaker for 3 d at 25°C. Spores were harvested by filtration through a miracloth, washed and their concentration was measured using a cell counter. Arabidopsis seedlings were grown on vertically placed, square plates (12 × 12 cm, Greiner) containing 1% agar supplemented with Murashige and Skoog (MS) medium and 1% sucrose for 15–16 d in a climate chamber (14 h : 10 h, light : dark cycle, 21°C). For *Fo* inoculation, seedlings were transferred into horizontally arranged square plates (12 × 12 cm) that contained a 2–3 cm strip of agar at one end, whereas the rest of the plate was filled with liquid MS medium containing 10^5^ Fo51756 spores ml^−1^. The shoots of the seedlings were placed onto the agar strip, to prevent direct contact with the fungal spores. The plates were closed, aluminium foil was placed above the root containing parts and the plates were placed in a climate chamber (11 h : 13 h, light : dark cycle, 28°C) for 6 d. At this stage, the *Fo*‐inoculated seedlings showed symptoms such as brown discoloration of the roots, and chlorosis of the leaf veins.

### RNA isolation and cDNA synthesis

Total RNA was isolated from roots of Fo5176‐inoculated Arabidopsis seedlings using TRIzol LS reagent (Ambion) according to the manufacturer’s instructions. DNA was removed by on‐column treatment with DNase (Thermo Scientific). cDNA was synthesised from 500 ng RNA using the RevertAid Reverse Transcription kit (Thermo Scientific) according to the manufacturer’s instructions.

### Streptavidin pulldown

Snap‐frozen roots of *Fo*‐inoculated seedlings were ground in liquid nitrogen and 100 mg was dissolved in 1.2 ml lysis buffer (50 mM Tris‐HCl pH 7.5, 150 mM NaCl, 10 mM EDTA, 10% glycerol, 10 mM DTT, 2% PVPP, 0.15% Nonidet P40 and protease inhibitor cocktail (Roche)). An aliquot of the supernatant was kept as input sample; the remaining material was incubated with 40 µl magnetic streptavidin beads (GE Healthcare, Chicago, IL, USA) and placed on a rotator for 2 h at 4°C. After washing three times, captured proteins were released by shaking the beads in 100 µl SDS loading buffer for 5–6 min at 96°C. As input, 20 µl of the lysate was loaded onto SDS gels, together with 10 µl of streptavidin‐captured fraction.

### Protein isolation and Western blot analysis

Proteins were isolated from *N. benthamiana* by grinding 20 mg leaf tissue in 100 µl lysis buffer (50 mM Tris‐HCl pH 7.5, 2% SDS, 5 mM DTT, and 1× protease inhibitor; Roche), and centrifuging at 16 000 ***g*** for 25 min. Proteins were separated by SDS‐PAGE using, in most cases, 13% gels. Western blotting was carried out using the semidry method on polyvinylidene difluoride (PVDF) membranes. Blots were probed with rat monoclonal anti‐HA antibodies (clone 3F10; Roche) at a dilution of 1 : 5000. The secondary goat‐anti‐rat antibody (Pierce) was used at a dilution of 1 : 8000. The signal was visualised with the ECL kits (GE Healthcare, ECL prime or Thermo Scientific, Super Signal West Pico) according to the manufacturer’s instructions.

## Results

### Determining the candidate effector repertoire of an Arabidopsis‐infecting *Fo* isolate

To gain insight into *Fo* effector‐mediated host manipulation, we set out to identify candidate effectors in strain Fo5176, which is pathogenic on Arabidopsis (Thatcher *et al.*, 2009). An effector prediction pipeline, encompassing 65 sequenced *Fo* isolates, revealed 62 candidates in this strain (van Dam *et al.*, 2016). Candidate effector prediction was based on the presence of a secretory signal peptide, a protein size of < 300 amino acids, and presence of inverted repeats of a transposable element in their promoter. Of the candidates, 19 showed a mosaic‐like phylogenic distribution; a hallmark of known *Fo* effectors (clusters C, D, E and F in van Dam *et al.*, 2016). This set was further refined by excluding genes that were mis‐annotated (1), are present in all pathogenic isolates (1), had an annotated function and/or domain (3), encoded a protein shorter than 60 amino acids (4) or presented an artefact of the prediction software (1). This resulted in a shortlist of nine candidate effectors, of which seven were successfully cloned and further characterised (Table S2). These seven candidate effectors include four *SIX* gene homologues present in Fo5176 (*Six1*, *Six4*, *Six8* and *Six9*) and three novel genes, designated *FoaEffector1*, *FoaEffector2* and *FoaEffector3* (from this point forward *Foa1*, *Foa2* and *Foa3*). In addition, one gene was included that was present in all the sequenced isolates, but showed high sequence variation, indicating that the gene may have undergone diversifying selection (*Foa4*).

**Fig. 1:**
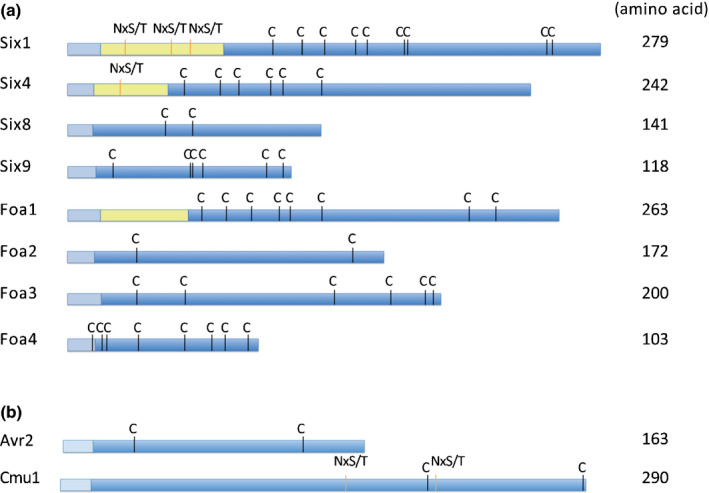
Candidate effectors of the *Fusarium oxysporum* 5176 isolate that is pathogenic on Arabidopsis. (a) Schematic representation of identified candidate effectors. (b) Exogenic effectors that were expressed in Fo5176 and are used in this study. Avr2 originates from a tomato‐infecting *Fo* isolate, while Cmu1 originates from the maize‐infecting fungus *Ustilago maydis*. Coloured boxes indicate: signal peptide (light blue), putative prodomain (yellow) and mature effector protein (blue). Cysteine residues (C) and predicted N‐glycosylation motifs (NxS/T) are indicated.

Fig. 1(a) shows a schematic representation of the identified candidate effectors. Without predicted signal peptides, their sizes ranged from 84 to 258 amino acids. All predicted proteins contain a paired number of cysteine residues, except Six1 (nine cysteines), suggesting that the ability to form disulfide bridges is a common feature for *Fo* effectors. The three largest candidate effectors, Six1, Six4 and Foa1, have a predicted N‐terminal prodomain that is separated from the rest of the protein by a predicted Kex2 protease cleavage site and is followed by a central region with six similarly spaced cysteines. In addition, the prodomains of Six1 and Six4 contain predicted N‐glycosylation motifs. Notably, Foa2 shares size and cysteine‐spacing with FolAvr2 and FomAvr2, two sequence‐unrelated effectors from tomato‐infecting and melon‐infecting *Fo* isolates, which are both recognised by intracellular NB‐LRR proteins (Ma *et al.*, 2013; Schmidt *et al.*, 2016). Six9, Foa3 and Foa4 are relatively cysteine‐rich, whereas Six8 has only two cysteine residues. Taken together, although the candidate Fo5176 effector sequences share potentially structural homologies with each other, as well as with other *Fo* effectors, this information does not reveal insights into their function or localisation during host colonisation by the fungus.

### Several *Fo5176* candidate effectors suppress the flg22‐ and/or chitin‐triggered ROS burst in *N. benthamiana*

**Fig. 2:**
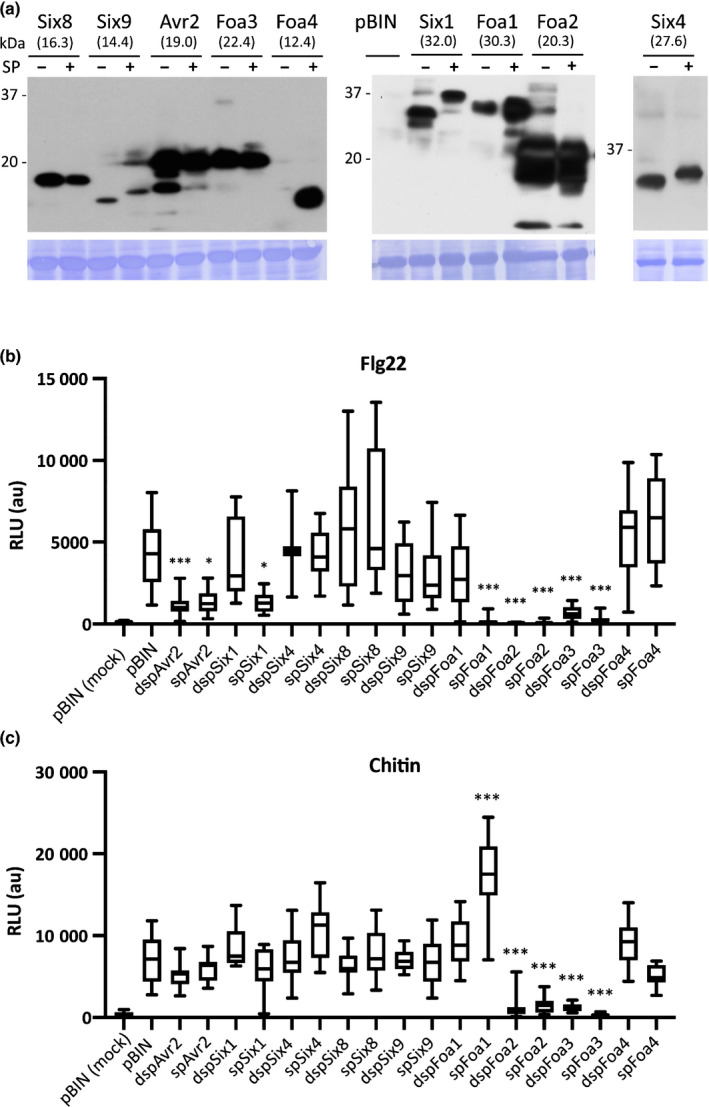
Several intracellularly or extracellularly acting *Fusarium oxysporum* effectors alter the flg22‐and chitin‐triggered ROS burst. (a) Western blots showing accumulation of candidate effectors expressed in *Nicotiana benthamiana* by agroinfiltration. Presence and absence of the signal peptide sequence in the plasmids used is indicated by (+) and (−), respectively. Molecular weight (kDa) markers are shown on the left. The predicted molecular sizes of the fusion proteins are indicated. Equal loading is verified by Coomassie staining of the blots. (b) Flg22‐triggered reactive oxygen species (ROS) generation in *N. benthamiana* leaves expressing the various candidate effectors. Presence and absence of their signal peptide is indicated by the prefix ‘sp’ and ‘dsp’. pBIN,  empty vector control containing an *Agrobacterium* strain carrying a binary vector without insert. Boxes extend from the 25^th^ to the 75^th^ percentile, whiskers from lowest to highest values, bar indicates the median; *n* = 16 leaf discs. (c) Chitin‐triggered ROS generation in *N. benthamiana* leaves expressing the various candidate effectors. Boxes extend from the 25^th^ to the 75^th^ percentile, whiskers from lowest to highest values, bar indicates the median; *n* = 16 leaf discs. Statistically significant differences to the flg22‐ and chitin‐treated empty vector controls are indicated (one‐way ANOVA: *, *P* < 0.05; ***, *P* < 0.001). The experiment was repeated twice with similar results. The second replicates are shown in Supporting Information Fig. S1b,c). Suppression of flg22‐triggered ROS by Foa1, Foa2 and Foa3 was observed in at least three independent experiments.

To elucidate a possible virulence function of the identified candidate effectors we tested whether they could suppress a classical PTI output, the ROS burst induced by flg22; a 22 amino acid derivative of the bacterial PAMP flagellin (Felix *et al.*, 1999). To this end, the candidate effector encoding genes were transiently expressed in *N. benthamiana*. To monitor whether the effectors act intracellularly or extracellularly, each candidate effector was expressed either with or without its endogenous signal peptide (indicated by the prefix ‘sp’ and ‘dsp’, for deleted sp). A C‐terminal HA tag allowed detection of protein accumulation by Western blot analysis. Transient expression of both sp and dsp versions resulted in comparable accumulation levels and similar apparent molecular sizes for most effector proteins (Fig. 2a). Only dspFoa4 did not accumulate to detectable levels, and the abundance of dspFoa1 was somewhat reduced as compared with the secreted version. Six1, Six4 and Six9 expressed with the signal peptide migrated at a higher molecular weights than variants lacking the signal peptide, indicative for a posttranslational modification. In conclusion, all *Fo* effector candidates were successfully produced *in planta*, enabling assessment of a potential involvement in flg22‐induced ROS burst in *N. benthamiana*.

Consistent with earlier reports, *N. benthamiana* leaves infiltrated with agrobacteria containing an empty binary vector responded to flg22 with a rapid and transient ROS burst (Fig. S1a; Zipfel *et al.*, 2006). As a positive control for effector‐mediated suppression we used FolAvr2, which acts intracellularly to inhibit various flg22–induced PTI responses, including the ROS burst (Di *et al.*, 2017). Indeed, as compared with the empty vector control, ROS levels were lower in the presence of Avr2 (Fig. 2b). Markedly, four out of the eight newly identified effector candidates also significantly reduced the flg22‐induced ROS response, whereby Foa1, Foa2 and Foa3 conferred a nearly complete suppression (Fig. 2b). Since Foa2 and Foa3 inhibited the flg22 output when expressed without their signal peptide, they likely act intracellularly. Surprisingly, both effectors suppressed the ROS response to the same extent when expressed with their cognate signal peptide, indicating that they either remain inside, or they re‐enter the cytosol after secretion. By contrast, Six1 and Foa1 only showed ROS suppression when expressed with their signal peptide, implying that these proteins acted extracellularly and have to be secreted to function (Fig. 2b).

Since flg22 is a bacterial PAMP and we used fungal effector protein candidates, we investigated whether these also altered responsiveness to the fungal PAMP chitin. Similar to flg22, chitin induced a rapid and transient ROS burst in *N. benthamiana* leaf discs infiltrated with agrobacteria containing an empty vector (Fig. S1b). As observed for the flg22 response, Foa2 and Foa3 strongly reduced chitin‐induced ROS production, indicating that these two effectors target a shared plant signalling component that is required to respond to both PAMPs (Fig. 2c). Interestingly spFoa1 showed an opposite effect; it suppressed flg22‐triggered, but enhanced chitin‐triggered ROS (Fig. 2b,c). In conclusion, while two intracellular *Fo* effectors robustly interfered with both flg22‐induced and chitin‐induced ROS production, the extracellular spFoa1 effector differentially affected the ROS response to these PAMPs.

Although clearly accumulating, Six4, Six8, Six9 and Foa4 did not alter flg22‐induced or chitin‐induced ROS production. However, a marked chlorosis was observed in *spSIX4*‐expressing leaf areas that was absent in sectors expressing *dspSIX4* or in the empty vector controls (Fig. S2). This finding suggests an extracellular activity of Six4, other than suppressing PTI. In conclusion, half of the investigated candidate effectors of the Arabidopsis‐infecting *Fo* strain negatively affected an early PTI signalling output in *N. benthamiana* leaves triggered by flg22 or chitin. One candidate triggers chlorosis, revealing a potential function in the host for these five proteins.

### Foa2 impairs mitogen‐activated protein kinase (MPK) activation and increases susceptibility to *Pst*

**Fig. 3:**
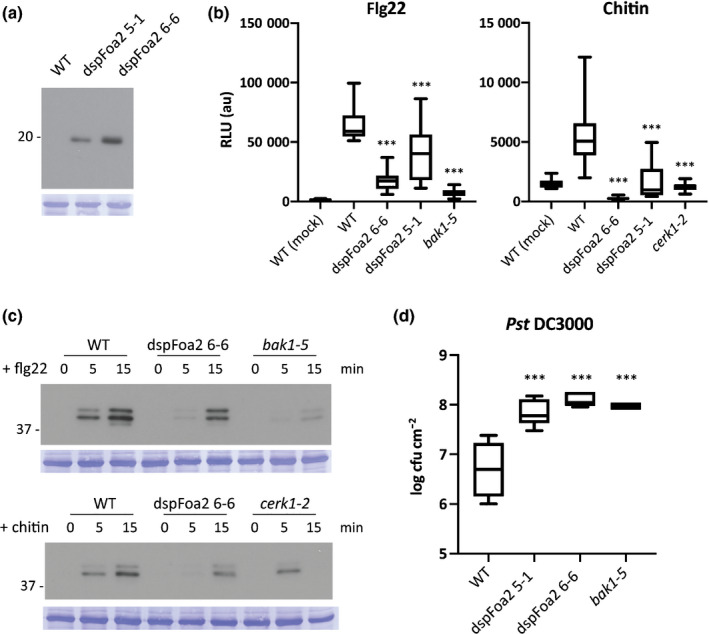
*dspFoa2*‐expressing Arabidopsis plants show diminished pathogen‐associated molecular pattern (PAMP) responses and enhanced pathogen susceptibility. (a) Anti‐HA Western blot revealing dspFoa2 accumulation in two independent transgenic lines. (b) Flg22‐triggered and chitin‐triggered reactive oxygen species (ROS) generation in *dspFoa2*‐expressing Arabidopsis plants. Statistically significant differences to the flg22‐ and chitin‐treated wild‐type controls are indicated (one‐way ANOVA: ***, *P* < 0.001). Boxes extend from the 25^th^ to the 75^th^ percentile, whiskers from lowest to highest values, bar indicates the median; *n* = 12 leaf discs. The experiment was repeated three times with similar results. (c) Flg22‐triggered and chitin‐triggered activation of mitogen‐activated protein (MAP) kinases in *dspFoa2*‐expressing Arabidopsis plants. Leaf discs were treated with elicitors for 0, 5 or 15 min and the accumulation of phosphorylated MAP kinases monitored by Western blot. Molecular weight (kDa) markers are shown on the left. Equal loading is verified by Coomassie staining of the blots. WT, wild‐type. (d) Five‐wk‐old Arabidopsis plants were spray inoculated with *Pst* DC3000, and bacterial titres were measured at 4 d post inoculation. Statistically significant differences to the wild‐type control are indicated (one‐way ANOVA: ***, *P* < 0.001). Boxes extend from the 25^th^ to the 75^th^ percentile, whiskers from lowest to highest values, bar indicates the median; *n* = 5. The experiment was repeated three times with similar results.

The transient expression screens in *N. benthamiana* revealed that multiple *Fo* effectors function as suppressors of early PTI signalling. To validate this finding, we focused on Foa2, which showed the strongest ROS burst suppression among the intracellular‐acting *Fo* effectors. Transgenic Arabidopsis plants were generated that stably expressed *dspFoa2*. Two independent, single insertion lines were selected and Western blotting revealed higher Foa2 levels in line 6‐6 than in 5‐1 (Fig. 3a). First, it was tested whether flg22‐triggered or chitin‐triggered ROS generation was altered in the *dspFoa2* Arabidopsis lines. Wild‐type plants showed a clear ROS burst, whereas *bak1‐5* and *cerk1‐2* did not respond to flg22 and chitin, respectively, confirming the specificity of the assay (Fig. 3b). Both *dspFoa2* lines showed a strongly reduced ROS accumulation in response to either PAMP as compared with wild‐type plants, in line with the findings of the transient expression assays (Fig. 3b). Moreover, the level of suppression seemed proportional to the effector accumulation, pointing to a dose‐dependent activity.

Activation of MPK cascades is a hallmark of early PAMP signalling (Couto & Zipfel, 2016). To assess whether Foa2 targets PTI outputs besides ROS generation, the flg22‐triggered and chitin‐triggered accumulation of phosphorylated MPKs was monitored by Western blot. Consistent with previous reports, two bands corresponding to MPK3 and 6 were detected in wild‐type plants (Saijo *et al.*, 2009). Their signal appeared 5 min after PAMP treatment and was further intensified after 15 min (Fig. 3c). The flg22 response was largely absent in *bak1‐5*, whereas *cerk1‐2* plants showed MPK activation after 5, but not after 15 min of chitin exposure, indicating residual responsiveness of this line. Importantly, MPK activation was markedly reduced in *dspFoa2* plants in response to both flg22 and chitin at both the 5 min and the 15 min time points (Fig. 3c). Altogether, these results showed that Foa2 inhibits both flg22 signalling and chitin signalling at the level of ROS production and MPK activation.

Next, we investigated whether the compromised PAMP responsiveness in dspFoa2 plants led to enhanced susceptibility to a pathogen. For this, Arabidopsis wild‐type, two *dspFoa2* lines and *bak1‐5* mutants were spray inoculated with the bacterial pathogen *P. syringae* pathovar *tomato* (*Pst*). At 4 d after inoculation bacterial titres were measured. In agreement with previous studies, *bak*1‐5 plants showed more than a 10‐fold higher bacterial count than wild‐type plants, this increase is in line with a compromised PTI response (Fig. 3d) (Schwessinger *et al.*, 2011). Importantly, both *dspFoa2* lines showed similarly increased bacterial titres as *bak1‐5*, suggesting that PTI is disabled by Foa2 (Fig. 3d). In summary, the stable transgenic *dspFoa2* lines revealed that this effector targets a component in PTI signalling that is required for at least two different PAMP receptors, resulting in enhanced pathogen susceptibility. Furthermore, this result showed that an effector candidate selected by its PTI‐suppressing activity in *N. benthamiana*, is also a potent PTI suppressor in the endogenous host of *Fo.*

### Host cell entry of *Fo* effectors during Arabidopsis root colonisation

The PTI‐suppressing activities of Six1 and Foa1 indicate that these two effectors act on an extracellular host target, whereas Foa2 and Foa3 function intracellularly and thus have to enter plant cells during root colonisation by the fungus. To investigate this further, we assessed whether intracellular accumulation of Foa2 and Foa3 – but not of Six1 and Foa1 – is detectable in *Fo* colonised Arabidopsis roots. For this we adjusted an assay in which biotinylation of Avi‐tagged effectors is monitored as a proxy for their presence inside living plant cells (Lo Presti *et al.*, 2017). The Avi effector tag combined an HA epitope for detection by Western blot with a biotin ligase recognition peptide motif that is biotinylated by the biotin ligase BirA, a recombinant bacterial enzyme stably expressed in transgenic Arabidopsis (Deal & Henikoff, 2011).

**Fig. 4:**
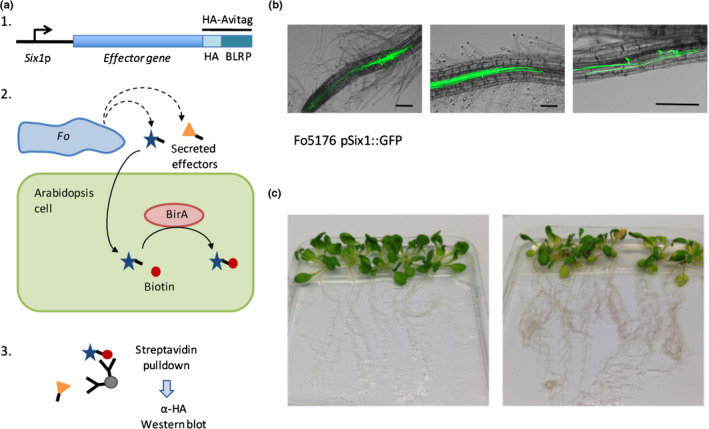
Strategy to detect the intracellular location of *Fusarium oxysporum* proteins in host cells upon colonisation of Arabidopsis roots. (a) Schematic representation of the *in planta* effector biotinylation assay. (1) The effector gene is placed under the control of the Six1 promoter. The chimeric protein is C‐terminal fused to the HA–AviTag. (2) Inoculation of Arabidopsis seedlings with a Fo5176 strain carrying the Effector–HA–Avi‐tagged construct. Avi‐tagged effectors that are translocated into host cells are biotinylated by BirA. (3) Western blot probed with anti‐HA antibodies detects biotinylated effectors that are captured by streptavidin beads from protein lysates from inoculated roots. (b) Arabidopsis roots 3 d post inoculation (dpi) with a Fo5176 strain that expresses a GFP reporter under control of the Six1 promoter. The green fluorescent GFP signal is visible in fungal hyphae colonising the root tip (left panel), the vasculature (middle) and root surface/outer tissues (right). Bars, 100 µm. (c) *In vitro*‐grown Arabidopsis seedlings 6 d after mock treatment (left panel) or inoculation with Fo5176 (right panel). Disease symptoms are visible in the Fo5176‐inoculated plants.

To compare uptake efficiency between different effectors *in vivo*, a synchronised infection and a relatively high amount of uniformly colonised roots are required. To generate such material, a semihydroponic system was developed in which sterile grown Arabidopsis seedlings were inoculated with *Fo*. First, we examined whether the fungus colonises the vasculature and causes typical disease symptoms in this setup. To monitor host colonisation a *Fo* reporter strain was used that carries a construct in which the promoter of the *Fo5176 SIX1* effector gene was fused to the coding sequence of GFP. The green fluorescence observed upon *Fo5176 pSix1::GFP* inoculation revealed successful fungal colonisation of the root tip, outer tissue layers and the vasculature of the plants (Fig. 4b). In line with the observed root colonisation, 5–7 dpi the seedlings developed clear disease symptoms such as root browning and leaf chlorosis starting at the veins, which was followed by tissue necrosis at later stages (Fig. 4c). These symptoms were comparable with those observed in soil‐grown plants inoculated with *Fo* (Thatcher *et al.*, 2009), validating the setup of the *in vitro* bioassay.

To assess effector uptake during root colonisation, Fo5176 strains expressing individual Avi‐tagged effectors were generated by *Agrobacterium*‐mediated transformation. As the *SIX1* promoter was found to be active throughout different stages of root colonisation (Fig. 4b), each effector was expressed from this promoter. In addition to the eight Fo5176 candidate effectors three controls were included: FolAvr2, which has been shown to function intracellularly (Houterman *et al.*, 2009; Di *et al.*, 2017), and two secreted *Fo* enzymes: a glycosyl hydrolase and an amidase that are both expected to function in the apoplast, thereby serving as negative controls for the uptake assay. *Fo* transformants were selected based on hygromycin resistance. Next, we tested whether tagged effectors were detectable in protein lysates of roots inoculated with the transformed fungi. For each construct three to five independent transformants were used to inoculate roots of Arabidopsis seedlings. Following protein isolation and separation on SDS‐PAGE, effector presence was detected by Western blotting and probing with an anti‐HA antibody. A specific band was detected for each tagged effector protein in at least one of the transformants, except for Six4 where none of the isolates showed a signal at the expected size (Fig. S3). In conclusion, except for Six4, the candidate effectors were readily detectable in protein extracts upon root colonisation by *Fo*, allowing assessment of their potential internalisation by the host cells.

To reveal which effectors were biotinylated during root colonisation – a proxy for their uptake and accumulation inside host cells – *BirA*‐expressing plants were inoculated with *Fo* strains producing Avi‐tagged effectors. Roots were harvested at 6 dpi when the first disease symptoms in the leaves started to emerge, but widespread tissue necrosis was not yet apparent. Protein lysates from inoculated roots were subjected to streptavidin pulldown assays, followed by anti‐HA Western blotting. An aliquot of the total lysate was blotted to verify accumulation of the tagged proteins in the root. To confirm that effector biotinylation solely relies on plant‐produced BirA, wild‐type Arabidopsis was inoculated with Fo5176 expressing Avi‐tagged Six8. As expected, only when isolated from BirA plants, and not wild‐type plants, Six8 showed binding to streptavidin (Fig. S4).

**Fig. 5:**
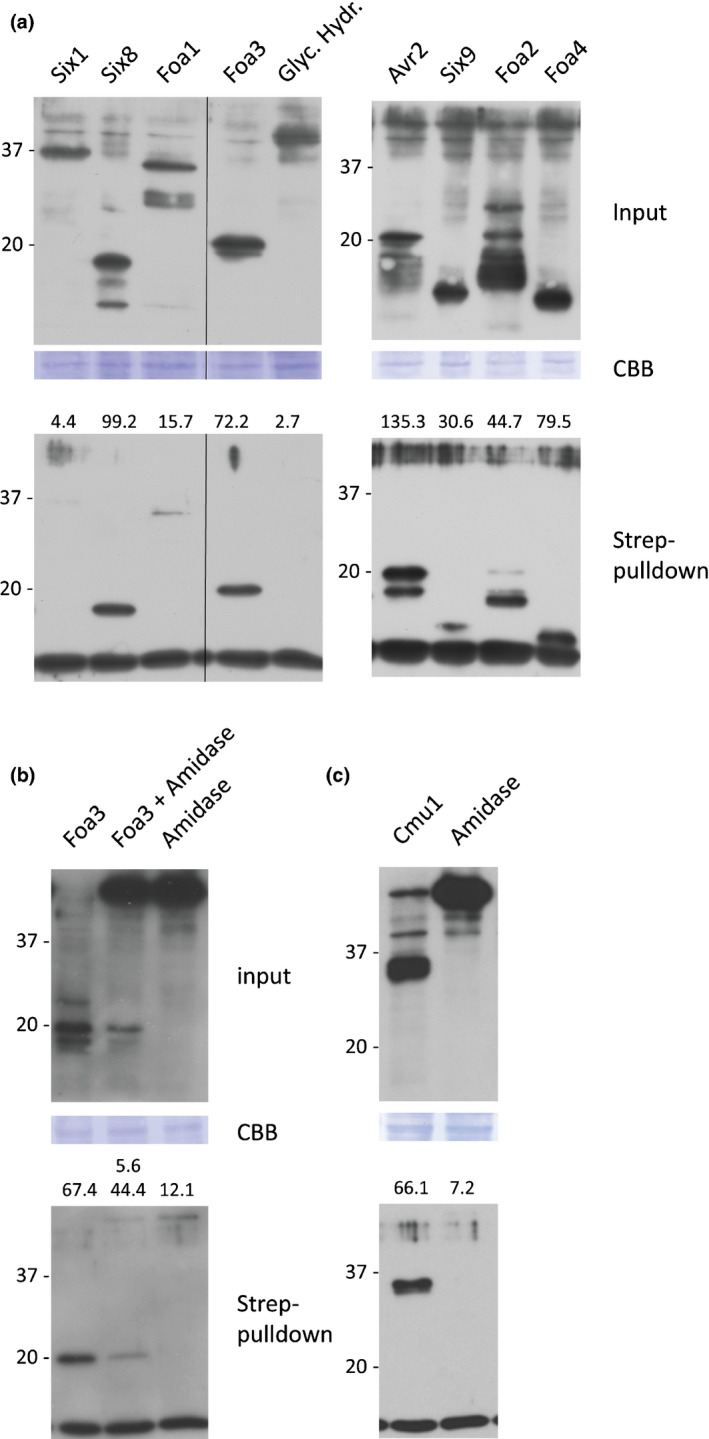
A subset of *Fusarium oxysporum* (*Fo*) effectors is biotinylated showing their uptake by host cells in *Fo*‐inoculated roots. (a) Col‐0 *BirA* seedlings inoculated with *Fo* transformants expressing Avi‐tagged effectors or enzymes (indicated above the blots). Roots from 15 or 16 seedlings were harvested at 6 d post inoculation (dpi) and biotinylated proteins were isolated from total protein lysates (top panel) using pulldown assays with magnetic streptavidin beads (lower panel). Protein blots were probed with anti‐HA antibodies. To allow direct comparison of the luminescence signals both blots were simultaneously exposed to the same film. The molecular weight (kDa) is indicated at the left side of each membrane. (b) Mixed and single inoculations with *Fo* transformants expressing the Avi‐tagged translocated effector Foa3 and the Avi‐tagged negative control amidase, followed by isolation of biotinylated proteins and anti‐HA Western blotting. (c) Inoculation with an *Fo* transformant expressing the *Ustilago maydis* effector Cmu1. The intensities of specific bands on both the input and pulldown blots were quantified and the band intensity after pulldown was normalised to the one in the input (shown above the lower blots, given as % of total). Of note, streptavidin pulldown samples are *c.* five‐fold enriched as compared with the input samples.

To compare the streptavidin pulldown efficiency between the different effectors and the controls, band signal intensities were quantified and their recovery after pulldown was normalised to the total accumulation levels of each protein (numbers shown above the streptavidin pulldown blots, Fig. 5a). Streptavidin beads effectively pulled down FolAvr2, whereas they did not pull down the negative control, the glycosyl hydrolase (Fig. 5a). This result showed that the assay is suitable to discriminate between host‐translocated and apoplastic proteins. In addition to FolAvr2, a clear signal upon streptavidin pulldown was detected for Six8, Foa3, Foa2 and Foa4, indicating their uptake by host cells (Fig. 5a). This result is in line with the intracellular PTI‐suppressing activity of Foa2 and Foa3, and with our finding that Six8 targets a plant transcriptional regulator (unpublished). The pulldown efficiency for Six9 was much lower than that of the putative cytoplasmic effectors, but higher than that of the apoplastic effectors, preventing to draw a firm conclusion on the potential localisation of this effector. Both presumed apoplastic effectors, Six1 and Foa1, showed a much weaker, yet detectable, signal after streptavidin pulldown (Fig. 5a). This signal could indicate that host cells internalised trace amounts of these two proteins, or that a minor fraction of extracellular protein was biotinylated, generating a weak nonspecific background signal. To address this concern, as an additional control mixed inoculations with strains expressing the translocated effector Foa3 and a negative control were performed. For this we used a secreted amidase from *Fo*, as this protein shows much higher total accumulation and thus provides a more suitable negative control. Although a weak signal after streptavidin pulldown was detected for the negative control, its signal intensity was much lower than for the translocated effector Foa3 (Fig. 5b). Altogether, the results of this translocation assay indicated that a subset of *Fo* effectors (Foa2, Foa3, Six8 and Foa4) accumulated inside host cells during infection, and that the putative apoplastic effectors (Six1 and Foa1) and extracellular enzymes did not, and remained extracellular.

### Protein size and charge do not explain effector internalisation

Based on the presented data, the putative Fo5176 effectors could be classified as either cytoplasmic or apoplastic. Next, we investigated whether features could be identified that are specifically associated with either category. A conserved sequence pattern comparable with the RxLR–dEER motif of oomycete effectors or the Y/F/WxC motif of powdery mildew effectors was not apparent in the cytoplasmically localised *Fo* effectors. Calculated isoelectric points ranged from 6.07 to 6.81 for the cytoplasmic effectors and from 5.49 to 8.75 for the apoplastic effectors. This overlap indicated that protein charge was also not associated with the ability of these proteins to enter plant cells (Table 1). Notably, all five *Fo* effectors that accumulated intracellularly were 20 kDa in size, or smaller, whereas the apoplastic proteins were significantly larger. This finding may imply that uptake in the *Fo* Arabidopsis interaction is restricted to small proteins. To test this hypothesis, we used the *Ustilago maydis* cytoplasmic effector Cmu1 (Djamei *et al.*, 2011), a 290 amino acid protein of similar size to the apoplastic *Fo* effectors Six1 and Foa1, in our root assay. Transgenic *Fo*5176 strains producing Avi‐tagged Cmu1 were generated and used in the effector translocation assay. When produced by *Fo* during plant infection Cmu1 clearly accumulated, and could effectively be pulled down by streptavidin, indicating successful host cell entry of the protein (Fig. 5c). As a negative control for host cell entry, uptake of the secreted amidase was assessed. Even though it also accumulated to high levels in *Fo‐*inoculated roots, the amidase was barely detected upon streptavidin pulldown, and thus mostly remained extracellular (Fig. 5c). Together, these results indicated that protein size *per se* does not determine effector uptake properties.

**Table 1:** General properties of cytoplasmic and apoplastic effectors and enzymes, after removal of their signal peptide.

Effector name	Number of amino acids	MW (kDa)	Calculated pI	Number of cysteines
Cytoplasmic				
Six8	122	13.0	6.15	2
Foa2	151	17.0	6.81	2
Foa3	176	19.1	6.44	6
Foa4	83	9.1	6.66	8
Avr2	144	15.7	6.07	2
Cmu1	269	29.8	6.00	2
Apoplastic				
Six1	258	28.7	8.57	9
Six4	225	24.3	6.47	6
Foa1	242	27.0	5.49	8
Glyc. hydrolase	364	38.2	8.75	9
Amidase	566	61.5	5.91	6
To be determined				
Six9	99	11.1	9.08	6

## Discussion

This study describes a systematic characterisation of effectors of an Arabidopsis root‐ and xylem‐infecting strain of *Fusarium oxysporum*. Four out of eight candidate effectors investigated were able to suppress the flg22‐induced or chitin‐induced ROS burst – an early PTI response – in leaves of *N. benthamiana*. This result implied that these *Fo* effectors acted on (a) target(s) that were present in both root and foliar cells and were evolutionary conserved between plant species. The observed redundancy among effector activity implied that targeting of PTI components is important for pathogenicity of this vascular pathogen. Furthermore, more than half of the studied effectors were found to function inside host cells, and, in correspondence, these could be biotinylated by a plant‐produced cytosolic BirA enzyme. Together, our findings show that several small proteins secreted by *Fo* during Arabidopsis root colonisation have properties that allow them to enter host cells and manipulate PTI.

Six1, Foa1, Foa2 and Foa3 from Fo5176 showed suppression of the flg22‐induced oxidative burst, a response that was detectable within minutes upon PAMP application, implying that *Fo* effectors intersected PTI signalling at an early step. The protein‐derived flg22 and the oligosaccharide chitin are perceived by different classes of receptors and co‐receptors, however their signalling converges on a subset of receptor‐like cytoplasmic kinases (RLCKs) (Couto & Zipfel, 2016). Thus, it is conceivable that the intracellularly acting Avr2, Foa2 and Foa3 effectors impaired early signalling by targeting the cytosolic part of receptor complexes and/or the associated RLCKs. By contrast, Foa1 and Six1 acted in the apoplast, thus their ability to interfere with PRR function must rely on a different mechanism. Previously investigated examples of apoplastically acting fungal effectors were *Cladosporium fulvum* ECP2 that sequesteres fungal chitin oligomers that are released by the activity of plant‐produced chitinases (de Jonge *et al.*, 2010), and Avr4 that prevents chitin degradation (van den Burg *et al.*, 2006). However, such a mechanism would not explain the effect of the apoplastic *Fo* effectors on flg22‐induced signalling. A putative mechanism on how they interfere with PAMP signalling is that these effectors target apoplastic proteases that process and activate secreted plant peptides called Rapid Alkalinisation Factors (RALFs) (Masachis *et al.*, 2016; Stegmann *et al.*, 2017). Currently, it is unknown why *Fo* possesses an effector (SpFoa1) that targets flg22‐induced, but not chitin‐induced, signalling. It is conceivable that this effector interferes with recognition of, yet unidentified, *Fo*‐derived PAMPs that share components with flg22 perception. Existence of at least one such PAMP was recently reported (Coleman *et al.*, 2019). Having identified a set of four sequence‐unrelated *Fo* effectors, acting at different locations and all counteracting PTI signalling, provides a unique set of molecular probes to dissect early immune signalling and to identify the proteins involved.

The observed redundancy of *Fo* effectors targeting the same process implied that interference with this process is a crucial element in host colonisation by this vascular pathogen. Intriguingly, a recent study revealed highly localised and damage‐dependent activation of PTI in Arabidopsis roots (Zhou *et al.*, 2020). Initial root surface colonisation by *Fo* is followed by its invasion of the xylem vessels that is likely to result in plant tissue damage and thus PTI activation, explaining the necessity for these PTI‐suppressing effectors. Whether these are produced and secreted in a highly localised manner during certain stages of the interaction is currently unknown.

Numerous fungal and oomycete effectors have been shown to function inside plant cells (Lo Presti *et al.*, 2015; Whisson *et al.*, 2016). However, demonstrating effector uptake during pathogen colonisation remains technically challenging. Lo Presti and colleagues developed an assay that detected effector biotinylation as a proxy for an intracellular protein location *in planta*. This assay was developed to analyse the localisation of effectors upon infection by the leaf‐invading pathogen *Ustilago maydis* (Lo Presti *et al.*, 2017). We adapted this method to elucidate which *Fo* effectors and/or enzymes were internalised into plant cells during root colonisation. Only a very weak signal indicating biotinylation of the two apoplastic effectors (Six1 and Foa1) and the two secreted enzymes (glycosyl hydrolase and amidase) was observed. It is unclear whether this finding reflects a small fraction of apoplastic proteins that entered host cells, or rather BirA leaking from damaged cells into the apoplast. Importantly, a much stronger biotinylation – indicating uptake – of the intracellular‐acting Avr2, Foa2, Foa3 and Six8 effectors was observed, supporting the specificity of the assay. In conclusion, the *in planta* biotinylation assay could be used to distinguish between intracellularly and extracellularly localised effectors of a fungal root pathogen. Whether *Fo* effectors are internalised by host cells from the xylem sap, from the root apoplast, or both, and whether close contact between the fungus and plant cells is required, as for instance in *Magnaporthe oryzae* (Khang *et al.*, 2010), remain questions for future research.

Another intriguing question is which features of effector proteins determine their uptake by plant cells. No apparent consistent difference in size, charge or amino acid composition (e.g. number of cysteine residues) was detected when comparing cytosolic and apoplastic effector proteins. Also, no obvious motifs such as RxLR, CRN or CHX were discernible in the translocated effectors, implying another mechanism directing their fate in the host. *M. oryzae* and *Phytophthora infestans* are proposed to use an unconventional, Golgi‐independent, secretory pathway for effectors that are destined for host cell entry (Giraldo *et al.*, 2013; Wang *et al.*, 2017). If *Fo* similarly uses a special secretory pathway for translocated effectors, then the information for entering this pathway must be embedded within the protein sequence, as all effectors were expressed from the same promoter and carry the same UTR sequences.

One mechanism resulting in an apparent specific uptake would be the selective retention of apoplastic proteins in the extracellular space, for instance by binding to the cell wall, and nonspecific uptake of all other proteins. Alternatively, translocated effectors might possess a special capacity that enable them to enter host cells. Our observation that all three intracellular‐acting effectors (Avr2, Foa2 and Foa3) suppress the flg22‐triggered ROS burst to a similar extent when expressed with or without their endogenous signal peptide are in support of this hypothesis. However, as these effectors do not share sequence homology nor contain obvious motifs or domains, their host cell entering activity might depend on hidden features in their structures, or alternatively they might use different entry mechanisms. Determining their 3D structures, as already done for the intracellularly acting Avr2 protein (Di *et al.*, 2017), might reveal shared folds or elements that correlate with the ability of effector proteins to enter host cells. The set of effectors acting at different locations in the host characterised here provides a good starting point for such a structural study. Understanding the mechanism of how *Fo* effectors target plant immunity and are translocated from the extracellular spaces into host cells, may provide new leads for strategies to combat fungal plant diseases.

## Author contributions

NT, MR and FLWT designed the research; NT and MP performed the experiments; NT and MP analysed the data; NT and FLWT wrote the manuscript.

## Supporting information

Please note: Wiley Blackwell are not responsible for the content or functionality of any Supporting Information supplied by the authors. Any queries (other than missing material) should be directed to the *New Phytologist* Central Office.

**Fig. S1** Flg22‐ and chitin‐triggered reactive oxygen species (ROS) generation in *N. benthamiana* leaves.
**Fig. S2** Expression of spSix4 leads to chlorosis in *N. benthamiana* leaves.
**Fig. S3** Accumulation of HA‐Avi‐tagged effectors in Arabidopsis seedlings inoculated with transgenic *Fusarium oxysporum* strains producing the proteins indicated.
**Fig. S4** Biotinylation of Six8 requires the plant‐produced BirA enzyme.
**Table S1** Primers used in this study.
**Table S2** Candidate effectors and enzymes.
