# Supplementary material for: The root‐invading pathogen Fusarium oxysporum targets pattern‐triggered immunity using both cytoplasmic and apoplastic effectors
Source: New Phytol. 2020 May 18;227(5):1479–92. doi: 10.1111/nph.16618 (PMC7496899; doi:10.1111/nph.16618)
Supplement: Supplementary file 1 — Fig. S1 Flg22‐ and chitin‐triggered reactive oxygen species (ROS) generation in N. benthamiana leaves. Fig. S2 Expression of spSix4 leads to chlorosis in N. benthamiana leaves. Fig. S3 Accumulation of HA‐Avi‐tagged effectors in Arabidopsis seedlings inoculated with transgenic Fusarium oxysporum strains producing the proteins indicated. Fig. S4 Biotinylation of Six8 requires the plant‐produced BirA enzyme. Table S1 Primers used in this study. Table S2 Candidate effectors and enzymes. [file NPH-227-1479-s001.pdf]

New Phytologist Supporting Information:

The root invading pathogen *Fusarium oxysporum* targets pattern-triggered immunity using both cytoplasmic and apoplastic effectors

Nico Tintor, Misha Paauw, Martijn Rep, Frank L. W. Takken

article acceptance date: 9 April 2020.

Fig. S1

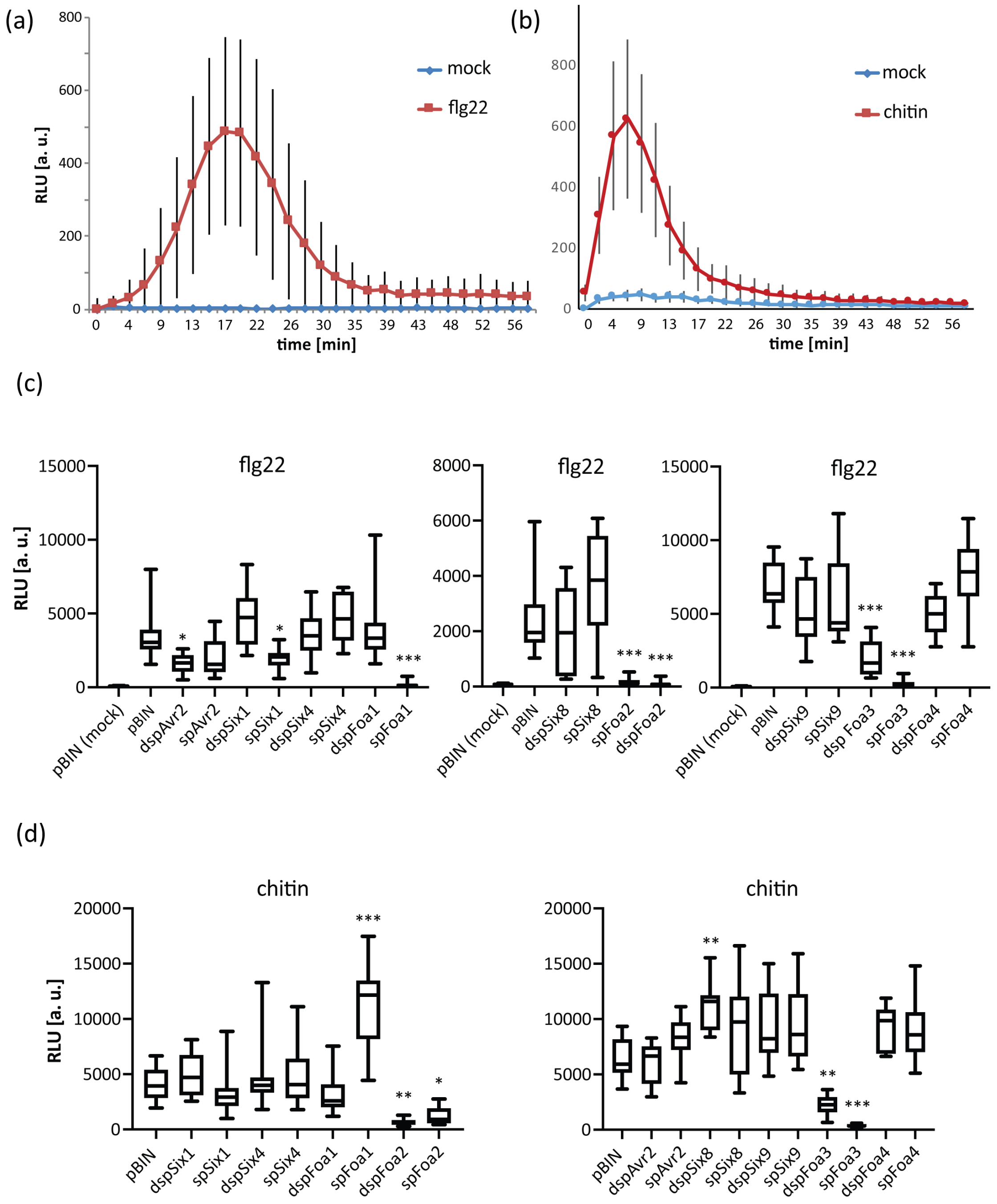

Fig S1 Flg22- and chitin-triggered reactive oxygen species (ROS) generation in *N. benthamiana* leaves

(a,b) Time course showing ROS generation in mock-, flg22- and chitin-treated *N. benthamiana* leaf discs. Each value represents the average of 16 leaf discs. Error bars indicate standard deviation.

(c) Flg22-triggered reactive oxygen species (ROS) generation in *N. benthamiana* leaves expressing the various candidate effectors. Presence and absence of their signal peptide is indicated by the prefix ‘sp’ and ‘dsp’. pBIN = empty vector control containing an *Agrobacterium* strain carrying a binary vector without insert.

(d) Chitin-triggered ROS generation in *N. benthamiana* leaves expressing the various candidate effectors. Statistically significant differences to the flg22- and

chitin-treated empty vector controls are indicated (one way ANOVA, (*) = P<0,05; (**) = p<0,01; (***) = p<0,001). Boxes extend from the 25th to the 75th percentile, whiskers from lowest to highest values, bar indicates the median; n = 16 leaf discs.

Fig. S2

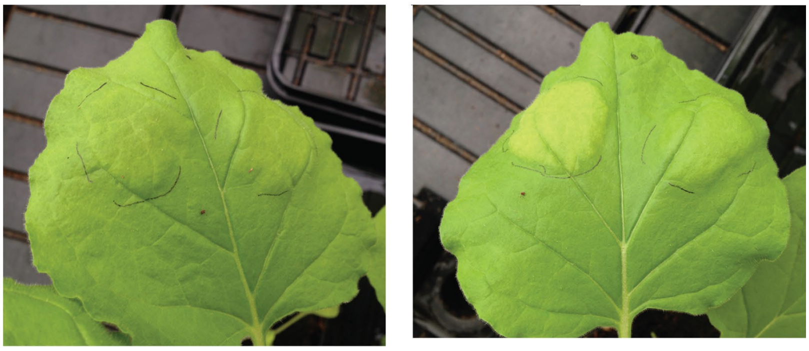

dspSix4 empty vector spSix4 empty vector

Fig S2 Expression of spSix4 leads to chlorosis in *N. benthamiana* leaves. Agrobacteria containing binary plasmids carrying either the *dspSix4* or *spSix4* gene, or an empty vector, were infiltrated into the encircled leaf areas. Pictures were taken 6 days post infiltration.

Fig. S3

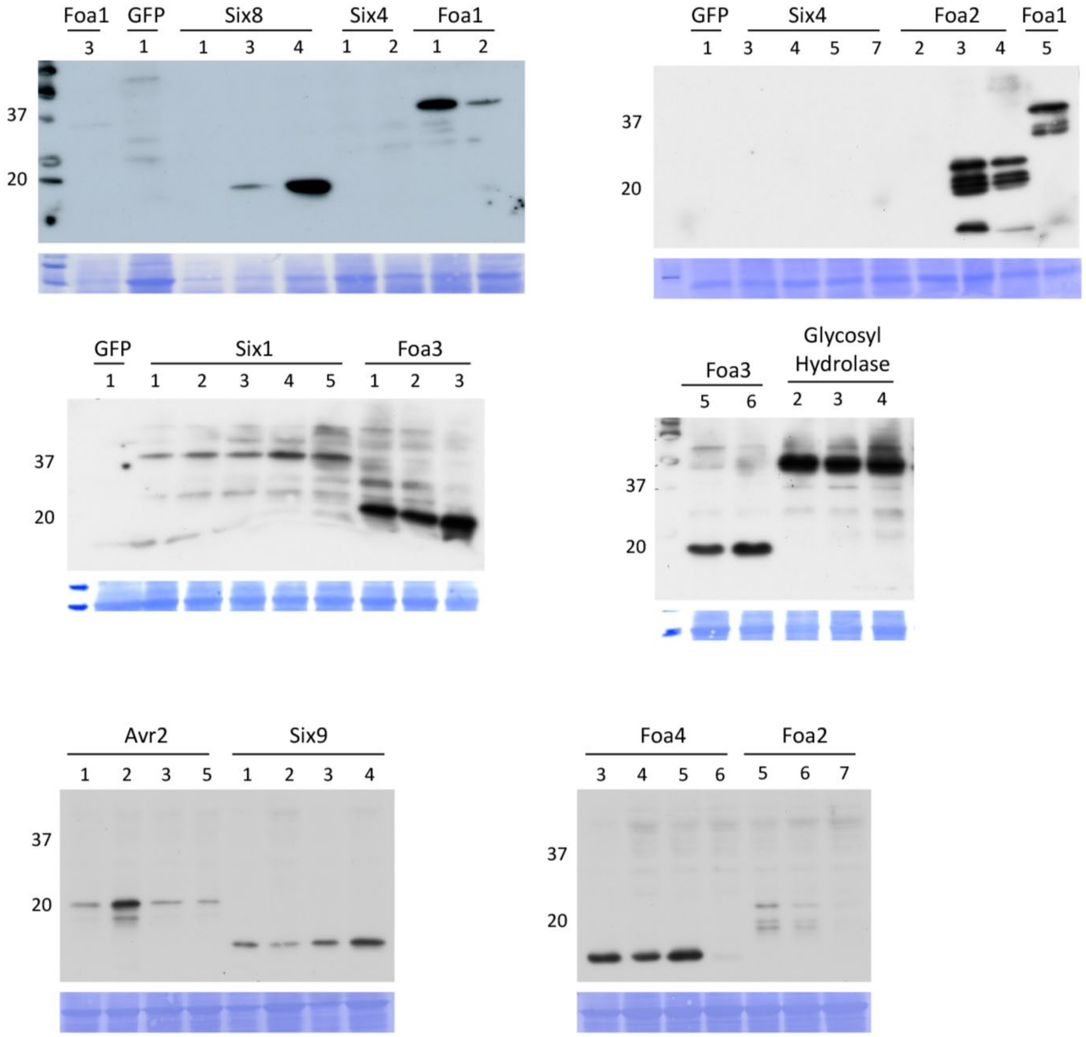

Fig S3 Accumulation of HA-Avi-tagged effectors in Arabidopsis seedlings inoculated with transgenic *Fusarium oxysporum* strains producing the proteins indicated.

Fo5176 was transformed with constructs encoding Avi-tagged effectors and -enzymes. Independent transformants were used to inoculate Arabidopsis seedlings. Five days post inoculation, proteins were extracted and analyzed by SDS-PAGE and Western blotting. Each lane contains proteins from seedlings inoculated with a specific *Fo* transformant (individual transformants are indicated by the numbers above the blots). Protein blots were probed with rat anti-HA antibodies. The molecular weight (kDa) marker is indicated at the left side of each membrane. Coomassie staining of the blots serves as loading control.

Fig. S4

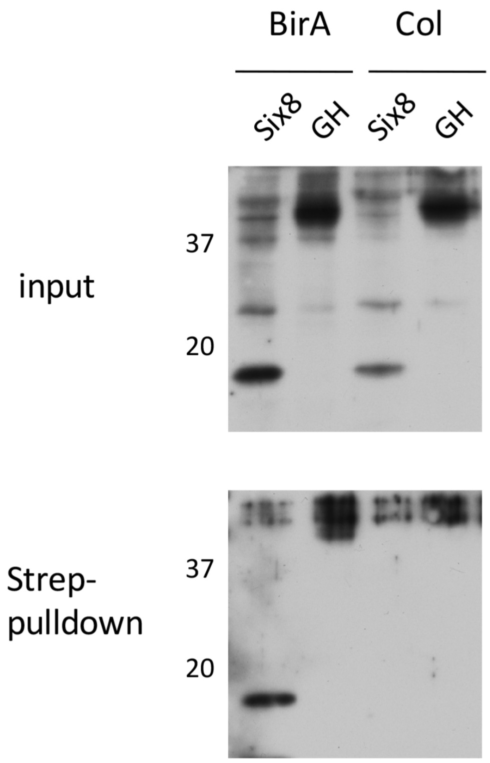

Fig S4 Biotinylation of Six8 requires the plant-produced BirA enzyme
Wildtype and BirA-containing Col seedlings were inoculated with Fo5176 strains that secrete Avi-tagged Six8 or Avi-tagged Glycosyl Hydrolase (GH). Roots were harvested at six days post inoculation. Biotinylated proteins were isolated from total protein lysates (top panel) using magnetic Streptavidin beads (bottom panel). Protein blots were probed with rat anti-HA antibodies. The molecular weight (kDa) marker is indicated at the left side of the membrane.

Table S1 Primers used in this study

Primer name	target gene	Sequence (5'-3')
HA-BLRP+	HA-Avi-tag	TTCTTGTCTAGAGGCGCGCCTACCCATATGACGTTCCAGATTACGCAGGACTTAATGACATCTTTGAGGCACAAAAGATCGAATGGCATGAGTAAAGATCTGTTGTT
HA-BLRP-	HA-Avi-tag	AACAACAGATCTTTACTCATGCCATTCGATCTTTTGTGCCTCAAAGATGTCATTAAGTCCTGCGTAATCTGGAACGTCATATGGGTAGGCGCGCCTCTAGACAAGAA
N036_HB_xma1	HA-Avi-tag with XmaI site	AACAACCCCGGGTTACTCATGCCATTC
N037_HB_xba1	HA-Avi-tag with XbaI site	TTCGACAAATCTAGAGGCGC
FoaSIX1pfor	Six1 promoter	ttcttgaagcttCGCGGCAATTCCTCTTGGAACATCG
FoaSIX1prev	Six1 promoter	taagaatctagaTTTGTCGAAAGCTCAAAATCC
**Primers for amplifying effectors and controls, including their signal peptide**		
SIX1-Asc1for	SIX1	ttctcaGGCGCGCCATGGCGCCCTATAGCATGG
SIX1-Asc1rev		tggaaaGGCGCGCCcGTAGGGGACATAGGGGCTGGT
SIX4-Asc1for	SIX4	ttctcaGGCGCGCCATGAATCTCAAGGCACTCG
SIX4-Asc1rev		tggaaaGGCGCGCCcAGCTAAGTTAAGTGTACCT
SIX8-Asc1for	SIX8	ttctcaGGCGCGCCATGCAACCCCTACGCGTTC
SIX8-Asc1rev		tggaaaGGCGCGCCcGAAATTGTTTATAAACTGGAC
N029_Six9a_for	SIX9	ttctcaGGCGCGCCATGAGGCTTTCTGCAGTTGC
N030_Six9a_rev		tggaaaGGCGCGCCcATTCCGGGTGCATTGTCCCA
Foa1_Fom6for	FOA1	ttctcaGGCGCGCCATGGTCGCCATCACTCTG
Foa1_Fom6rev		tggaaaGGCGCGCCcAGATCTCAAGATGTCATAG
N019_Foa2_for	FOA2	ttctcaGGCGCGCCATGTTCTTCATTAAACCAATC
N020_Foa2_rev		tggaaaGGCGCGCCcCATGTCTCTAATAATATACG
N023_Foa3_for	FOA3	ttctcaGGCGCGCCATGGAGTCTTACAAAAGTCTG
N024_Foa3_rev		tggaaaGGCGCGCCcAAATTTGCCACAACCCAGTTGG
N025_Foa4_for	FOA4	ttctcaGGCGCGCCATGAAGGTTTCACTTCTCGC
N026_Foa4_rev		tggaaaGGCGCGCCcAGGAAACTTCAGTCGGCAGC
N015_GH_for	Glycosyl Hydrolase	ttctcaGGCGCGCCATGCGCTTTTCACCCCTTCT
N016_GH_rev		tggaaaGGCGCGCCcGGTGCAGAATGCACCAGCAGG
N041_Avr2HB_for2	AVR2	tcaGGCGCGCCATGCGTTTCCTTCTGCTTATAGC
N054_A2HB_r2		taaGGCGCGCCcATCCTCTGAGATAGTAAGATAG
N106_Cmu1_F	CMU1	tcaGGCGCGCCatgaagttgagcgtgtccatc
N107_Cmu1_R		aaaGGCGCGCCcggtgcacttgttggcgtggt
N104_Amd_F	Amidase	tcaGGCGCGCCatgaagcttctcgggttgtcg
N105_Amd_R		aaaGGCGCGCCcaaagggcacgggaacgttacg
**Primers for amplifying effectors and controls, excluding their signal peptide**		
N049_Six1dSP	SIX1	ttctcaGGCGCGCCATGCAAGAGGCTGCGGTTGGGGA
N050_Six4dSP	SIX4	ttctcaGGCGCGCCATGCTTCCAAAGGGGGAGGAGG
N051_Six8dSP	SIX8	ttctcaGGCGCGCCATGACGCATATTGATAAGAGCC
N055_Six9dSP	SIX9	ttctcaGGCGCGCCATGCAGAATAGGAATATTCAAGTTGG
N052_Foa1dSP	FOA1	ttctcaGGCGCGCCATGTCGCCTGCCGAAGTCGTACC
N056_Foa2dSP	FOA2	ttctcaGGCGCGCCATGAAGCCCTTACCTGAGAAATCTG
N057_Foa3dSP	FOA3	ttctcaGGCGCGCCATGGTGGAATACAAGTGGGTTTTCACC
N058_Foa4dSP	FOA4	ttctcaGGCGCGCCATGAGCTACAGCAAATGCCATTG
N048_Avr2dSP	AVR2	ttctcaGGCGCGCCATGCTACCTGTGGAAGATGCC

Table S2 Candidate effectors and enzymes

**Name**	**GeneBank ID**	**origin**	**Nr. Amino acids**	**Nr. Cys residues**	**SignalP_score**	**SP_size**	**SP_seq**
Six1	FOXB_01734	*Fo5176*	279	9	0.772	21	mapysmvllgalsilgfgaya
Six4	FOXB_04209	*Fo5176*	242	6	0.648	17	mnlkalvviasvavtsa
Six8	FOXB_13262	*Fo5176*	141	2	0.821	18	mqplrvlllfplavsvaa
Six9	FOXB_17801	*Fo5176*	118	6	0.544	19	mrlsavaatafaifstaea
Foa1	FOXB_16439	*Fo5176*	263	8	0.776	21	mvaitlkvlagvaaflaivna
Foa2	FOXB_15742	*Fo5176*	172	2	0.682	20	mffikpifvafsfyialita
Foa3	FOXB_16928	*Fo5176*	200	6	0.885	23	mesykslmsllllfthlahlaqa
Foa4	FOXB_14349	*Fo5176*	103	8	0.803	19	mkvsllallalstcasaca
Avr2	FOXG_16398.3	*Fol*	163	2	0.886	19	mrfllliamsmtwvcsiag
Glycosyl Hydrolase	FOXB_15917	*Fo5176*	381	9	0.518	17	mrfspllfgsfiasafa
Amidase	FOXG_11632	*Fol*	585	6	0.71	19	mkllglslatgliaqgvsa
Cmu1	UMAG_05731	*Ustilago maydis*	290	2	0.784	21	mklsvsifvllavsafgggsa
